# Insulin metabolism markers are predictors of subclinical atherosclerosis among overweight and obese children and adolescents

**DOI:** 10.1186/s12887-018-1347-9

**Published:** 2018-11-23

**Authors:** Golaleh Asghari, Pooneh Dehghan, Parvin Mirmiran, Emad Yuzbashian, Maryam Mahdavi, Maryam Tohidi, Tirang R. Neyestani, Farhad Hosseinpanah, Fereidoun Azizi

**Affiliations:** 1 Department of Clinical Nutrition and Dietetics, Faculty of Nutrition Sciences and Food Technology, National Nutrition and Food Technology Research Institute, Shahid Beheshti University of Medical Sciences, Tehran, Iran; 2 Department of Imaging, Research Development Center, Taleghani Hospital, Shahid Beheshti University of Medical Sciences, Tehran, Iran; 3 Nutrition and Endocrine Research Center, Research Institute for Endocrine Sciences, Shahid Beheshti University of Medical Sciences, P.O. Box: 19395-4763, Tehran, Iran; 4 Nutrition and Endocrine Research Center, Research Institute for Endocrine Sciences, Shahid Beheshti University of Medical Sciences, Tehran, Iran; 5 Obesity Research Center, Research Institute for Endocrine Sciences, Shahid Beheshti University of Medical Sciences, Tehran, Iran; 6 Prevention of Metabolic Disorders Research Center, Research Institute for Endocrine Sciences, Shahid Beheshti University of Medical Sciences, Tehran, Iran; 7 Laboratory of Nutrition Research, National Nutrition and Food Technology Research Institute, Faculty of Nutrition Sciences and Food Technology, Shahid Beheshti University of Medical Sciences, Tehran, Iran; 8 Obesity Research Center, Research Institute for Endocrine Sciences, Shahid Beheshti University of Medical Sciences, P.O. Box: 19395-4763, Tehran, Iran; 9 Endocrine Research Center, Research Institute for Endocrine Sciences, Shahid Beheshti University of Medical Sciences, Tehran, Iran

**Keywords:** Insulin resistance, Atherosclerosis, Children, Adolescents, obesity

## Abstract

**Background:**

To investigate the association between markers of insulin metabolism and carotid intima-media thickness(cIMT) among overweight and obese children and adolescents.

**Methods:**

A total of 378 children and adolescents aged from 6 to 13 years, with WHO body mass index Z-Scores ≥2 were enrolled in this study. We measured fasting serum insulin and glucose, conducted a homeostatic model assessment of insulin resistance(HOMA-IR), and calculated the quantitative insulin sensitivity check index(QUICKI). Carotid intima-media thickness was measured in the common carotid artery with high-resolution ultrasonography.

**Results:**

The study participants consisted of 198 boys and 180 girls with a mean(±SD) age of 9.3 ± 1.7 years, 18.3% being pre-pubertal. In boys, after controlling for confounders, a one-SD increase in fasting insulin and HOMA-IR were associated with 0.351 mm(*P* < 0.001) and 0.350 mm(*P* < 0.001) increases in cIMT, respectively. However, a one-SD increase in QUICKI was associated with a − 0.305 mm(*P* = 0.001) decrease in cIMT. When categorizing into tertiles, a one-SD increase in fasting insulin and HOMA-IR were associated with 87 and 81% increases in the odds of higher categories of cIMT(both *P* < 0.05). However, a one-SD increase in QUICKI was associated with 37% lower odds of higher categories of cIMT(*P* = 0.022). No significant associations were found among girls.

**Conclusion:**

This study demonstrated that insulin resistance and sensitivity markers were independent predictors of cIMT in overweight and obese boys, but not in girls, highlighting the importance of chronically elevated insulin levels for predisposing these boys to alterations in their vascular structure.

## Background

Obesity-related insulin resistance and chronically elevated insulin concentrations that begin in childhood can induce atherothrombotic mechanisms, reduce fibrinolytic balance, and impair endothelial function, which independently contributes to future cardiovascular events in adulthood [1, 2]. Atherosclerosis develops gradually, over the course of a lifetime, and a long crucial phase of the process consists of several silent dysfunctional changes of the endothelium [3]. The increased mortality associated with atherosclerosis is related to the duration and intensity of risk factors presenting throughout a lifetime. Therefore, to prevent cardiovascular disease from manifesting and known factors leading to clinical events, it is important to unravel which factors play a role in the earlier and later stages of the atherosclerotic disease.

In recent years, high-resolution B mode ultrasonography has been frequently used in children and adolescents to measure carotid intima-media thickness (cIMT) for the purpose of assessing the presence of subclinical atherosclerosis. Atherosclerotic monitoring with IMT is both sensitive and reproducible, providing a surrogate measure of atherosclerotic burden and cardiovascular risk which may, in turn, serve as possible predictors of future cardiovascular events [4, 5]. Previous observational studies indicated that IMT in children and adolescents elevated with familial hypercholesterolemia [6], type 1 diabetes mellitus [7], and hypertension [8]. Measuring cIMT in children and adolescents with obesity has been recommended by the Association for European Paediatric Cardiology (AEPC), as it is capable of recognizing early vascular alterations and thus prevent myocardial infarction and stroke [9].

With regard to a possible relationship between IMT and insulin resistance/hyperinsulinemia in children and adolescents, the results remain inconsistent, with some studies reporting an adverse relation between insulin resistance and vascular measures [10, 11], while others observed no significant relations at all [12–14]. The studies in question did not look for differences based on gender or pubertal status.

By studying possible associations between insulin resistance and measures of vascular structure, while considering gender and pubertal maturation status, we may gain some insight on and advance our understanding of the complex gender-specific relations between obesity and vascular structure in response to insulin level. The present study has addressed this question by investigating possible associations between markers of insulin metabolism and cIMT among children and adolescents with either overweight or obesity.

## Methods

### Study population

This study was conducted during the baseline (cross-sectional) phase of a randomized, double-blind trial, aimed at investigating the effect of different doses of one-year vitamin D supplementation on cIMT and other bone and cardiovascular variables in children and adolescents with an age- and sex-specific body mass index (BMI) Z-score ≥  1 (according to the criteria established by the World Health Organization). Participants, aged 6 to 13 years, were recruited from primary schools located in three Tehran (Iran) districts. The baseline screening took place between June 2016 and March 2017. Individuals were eligible for inclusion if they had no known medical illnesses such as diabetes, liver, or kidney diseases, were not taking any dietary supplements, or using pharmaceutical agents that affect glucose and lipid metabolism. A total of 378 participants met these selection criteria and were enrolled at the baseline. The sample size for the trial was determined by a sample size calculation designed to achieve 80% power to detect a 0.02 mm difference in cIMT with a 0.05 standard deviation (95% confidence interval) between different groups of the original trial.

### Measurements

Fat and soft lean mass were assessed by portable bioelectrical impedance analyzer GAIA 359 PLUS 8-contact electrode bioelectrical impedance (BIA) system (Jawon Medical Co. Ltd., Shinsang, Korea). During the measurement, all participants removed their shoes and socks, wore light clothing, and stood with the soles of their feet making contact with the foot electrodes, while holding the hand electrodes in their bare hands. All necessary variables including sex, height, and age, were input into the instrument. The BIA system measured the impedance via a tetra-polar multi-frequency (5, 50, 250 kHz) electrode method. Resistance to the 500 μA current was measured while the participants stood motionless on the analyzer’s platform, their arms raised about 30°, while lightly holding onto the hand electrodes. For the data interpretation, we used the manufacturer’s software in its “standard” setting.

Body weight was measured using the scale function of the GAIA 359 PLUS (100-g accuracy) while the subjects were standing barefoot and wearing light clothing. Height was measured while participants were standing without shoes and shoulders in normal alignment, using a stadiometer with an accuracy of 0.5 cm. Body mass index (BMI) was calculated as weight (in kilograms) divided by height (in meters) squared (kg/m^2^). Waist circumference (WC) was measured down to the nearest 0.5 cm at the level of the umbilicus, over light clothing, and without any pressure, using a tape meter.

Blood pressure was measured twice, at least 1 min apart, using a mercury sphygmomanometer and the Korotkoff sound technique, with an accuracy of 2 mmHg, after participants had been resting for 15 min on a chair; the average of both measurements served as the participant’s final pressure; systolic/diastolic blood pressures (SBP/DBP) were determined from the first onset/disappearance of sound.

Blood samples were collected from all study participants between 8:00 and 10:00 AM, after 10–12 h of overnight fasting. All the blood analyses were carried out at the RIES research laboratory. Fasting blood glucose (FBG) was measured by enzymatic colorimetric method using glucose oxidase (Pars Azmoon, Tehran, Iran) and the Selectra 2 auto-analyzer (Vital Scientific, Spankeren, Netherlands) with intra- and inter-assay coefficients of variation (CVs) of 1.1 and 1.4%, respectively. Fasting serum insulin was determined by the electrochemiluminescence immunoassay (ECLIA) method, using Roche Diagnostics kits and the Roche/Hitachi Cobas e-411 analyzer (Roche Diagnostics, GmbH, Mannheim, Germany). Intra- and inter-assay CVs were 1.3 and 2.5%, respectively. To monitor the quality of measurements, assayed serum controls in different concentrations were used for the glucose (Pars Azmoon, Tehran, Iran) and insulin assays (Lyphochek Immunoassay Plus Control, Bio-Rad Laboratories).

Physical activity was assessed using the Modifiable Activity Questionnaire (MAQ) to calculate metabolic equivalent task minutes per week; the high reliability (97%) and moderate validity (49%) of the Persian translated MAQ in adolescents have been ascertained previously [15].

Pubertal status was classified according to the Tanner scale by a well-trained endocrinologist, dividing the participants into 2 groups based on breast and genital stages: pre-pubertal (boys at genital stage I, girls at breast stage I) and pubertal (boys at genital stage ≥II, girls at breast stage ≥II).

Carotid intima-media thickness was measured by a well-trained radiologist (P.D.). Participants were examined in the supine position with the head slightly extended and rotated away from the examiner. The carotid arteries were interrogated using a high-resolution Samsung ultrasound machine (model UGEO WS80A) with a linear-array transducer operating at a frequency of at least 7 MHz. Depth, gain, and focus was adjusted for each participant individually so that the arterial lumen was completely anechoic and in the center of the image. Common cIMT was measured from longitudinal B-mode images of the distal 1 cm of the far wall of each common carotid artery (CCA), between the intimal-luminal and the medial-adventitial interfaces of the carotid artery wall, represented as a double-line density on the ultrasound image. In rare cases where appropriate images of the distal CCA could not be obtained, proximal or mid-CCA images were used for IMT measurement. Measurements were performed using the automated edge-tracking software (automated IMT calculator) which obviated the need to perform manual measurements.

### Definitions

Insulin resistance/sensitivity was calculated as follows:

Homeostatic model assessment of insulin resistance (HOMA-IR) = [fasting insulin (μU/ml) × fasting glucose (mmol/l)]/22.5;

Quantitative insulin sensitivity check index (QUICKI) = 1/[log fasting insulin (μU/ml) + log fasting glucose (mg/dl)].

Abdominal obesity was defined as waist circumference (WC) ≥ 90th percentile for age and sex, according to national reference curves [16].

### Statistical analysis

Quantitative variables were expressed as the mean ± SD or median (interquartile range) while categorical variables were expressed as percentages. Continuous and categorical variables were compared according to sex using a t-test and a Chi square-test, respectively. Multiple linear regression analysis was used to evaluate the association of markers of insulin metabolism and cIMT. A proportional odds model (POM) was used to test for a possible association between an insulin metabolism marker increase of approximately 1 SD and tertiles of cIMT using the ranges: cIMT< 0.38, 0.38 ≤ cIMT< 0.42, and cIMT≥0.42 mm. The proportional odds assumption was assessed through a chi-squared score test for each covariate to examine whether the assumption was violated. Proportional odds assumptions were generally appropriate. To test whether pubertal stage or gender could affect the contribution of fasting insulin, the interaction terms for [female × fasting insulin] and [pre-pubertal × fasting insulin] were used as separate inputs in our multivariable linear regression models. The likelihood ratio test for interaction terms was not significant. Nevertheless, the interaction term for gender was of biological importance which is why each model was performed in the subset specified by gender. Waist circumference, body fat percentage, physical activity, and Tanner stages were considered as confounding variables. Collinearity was assessed using Pearson’s rank correlation coefficient and variance inflation factor. No collinearity was found between body fat percentage and WC. All analyses were performed using IBM SPSS for Windows, version 20 (SPSS, Chicago, IL, USA) with the significance level set at *P* < 0·05 (two-tailed).

## Results

The study consisted of 378 participants (198 boys and 180 girls) with a mean (±SD) age of 9.3 ± 1.7 years and 18.3% in the pre-pubertal stage. Compared to girls, boys had a higher physical activity, more hours of TV watching, a greater BMI, WC, soft lean mass, prevalence of obesity, a lower fat mass and HOMA-IR (*P* < 0.05, Table 1).

**Table 1:** Characteristics of 378 overweight and obese children and adolescents

	Total (*n* = 378)	Girls (*n* = 180)	Boys (*n* = 198)	*P*-value
Age (years)	9.3 ± 1.7	9.2 ± 1.7	9.4 ± 1.8	0.326
Pre-pubertal (%)	18.3	15.0	21.3	0.113
Physical activity (MET/hr./wk)	13.3 (5.1–31.7)	8.9 (3.3–17.9)	20.1 (7.9–41.2)	< 0.001
Watching TV (hour/d)				
≤ 1	14.1	14.2	13.9	0.024
2–3	38.6	45.5	32.5	
≥ 4	47.3	40.3	53.6	
Height (cm)	139.7 ± 11.0	138.9 ± 10.9	140.5 ± 11.0	0.142
Z-Score Height	0.86 ± 1.00	0.80 ± 1.07	0.91 ± 0.93	0.291
BMI (kg/m^2^)	23.3 ± 3.4	22.8 ± 3.2	23.8 ± 3.4	0.004
Z-Score BMI	2.55 ± 0.73	2.34 ± 0.83	2.74 ± 0.83	< 0.001
Obese (%)	68.8	55.6	80.8	< 0.001
Waist circumference (cm)	80.8 ± 9.5	79.4 ± 8.9	82.0 ± 10.0	0.009
Abdominal obesity (%)	92.6	93.1	92.1	0.705
Fat mass (%)	26.3 ± 6.9	28.6 ± 4.2	24.2 ± 8.1	< 0.001
Soft-lean mass (kg)	30.7 ± 7.2	29.1 ± 6.6	32.2 ± 7.3	< 0.001
Fasting glucose (mg/dl)	90.7 ± 9.3	91.5 ± 8.6	89.9 ± 9.9	0.103
Fasting insulin (μU/ml)	12.4 (8.5–19.0)	12.9 (9.3–20.6)	11.9 (7.9–18.2)	0.085
HOMA-IR	2.80 (1.92–4.34)	3.00 (2.02–4.56)	2.66 (1.67–4.13)	0.048
QUICKI	0.333 ± 0.040	0.328 ± 0.031	0.338 ± 0.046	0.023
Carotid intima media thickness (mm)	0.40 ± 0.057	0.40 ± 0.004	0.41 ± 0.060	0.100

*BMI* body mass index, *HOMA-IR* homeostatic model assessment of insulin resistance, *QUICKI* quantitative insulin sensitivity check index

Quantitative variables were expressed as the mean ± SD or median (interquartile range) and categorical ones as percentages

Abdominal obesity was defined based on national age- and sex-specific 90th percentile (1)

**Fig. 1:**
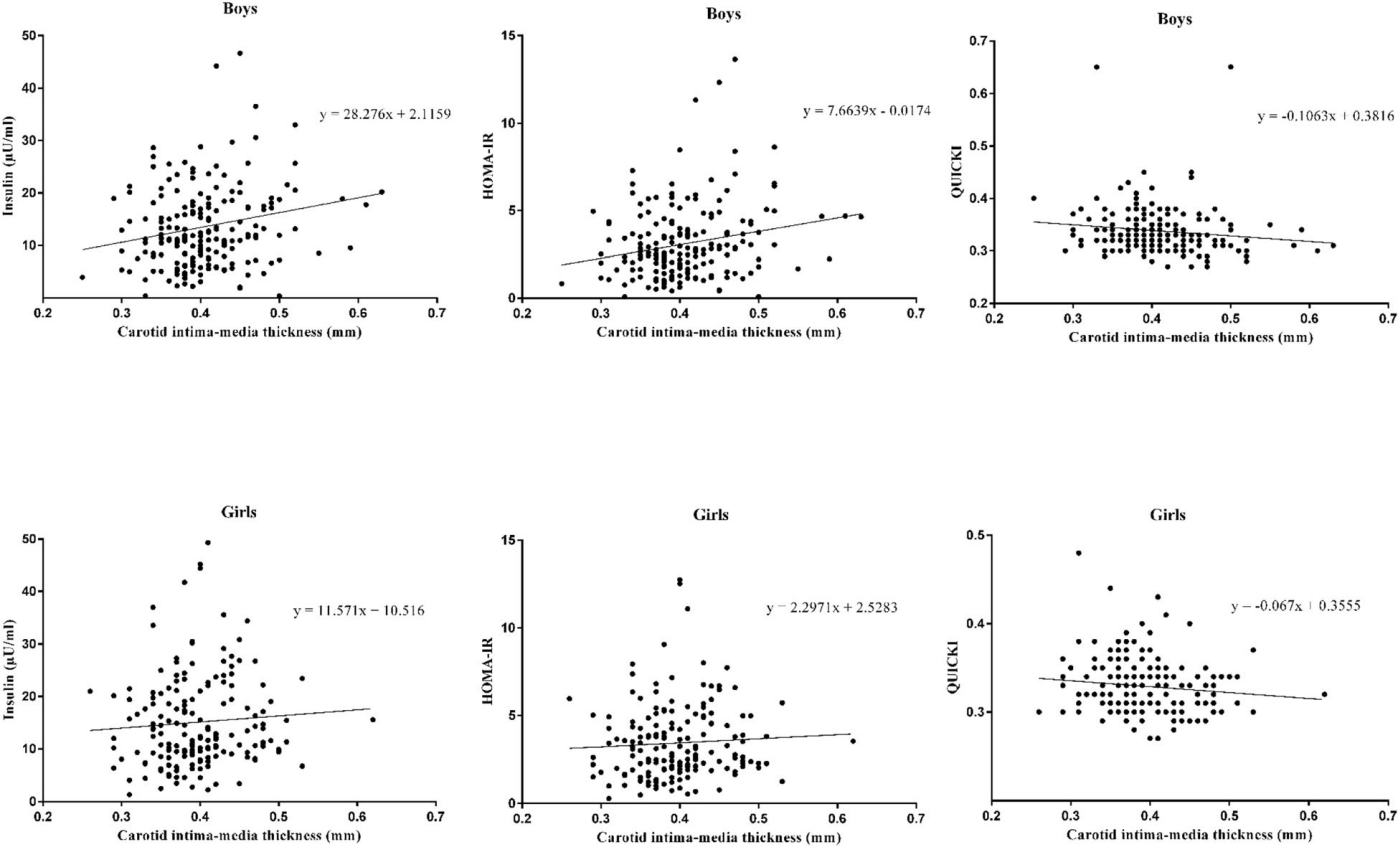
Gender-specific correlations of fasting insulin, homeostatic model assessment of insulin resistance (HOMA-IR), and quantitative insulin sensitivity check index (QUICKI) with carotid intima-media thickness among 378 overweight and obese children and adolescents

In boys, but not girls, both fasting insulin and HOMA-IR were positively, and QUICCKI negatively correlated with cIMT (Fig. 1). In boys, after controlling for body fat mass percentage, WC, physical activity, and Tanner stage, a one-SD increase in fasting insulin and HOMA-IR were associated with 0.351 mm (*P* < 0.001) and 0.350 mm (*P* < 0.001) increases in cIMT, respectively. However, a one-SD increase in QUICKI was associated with a − 0.305 mm (*P* = 0.001) decrease in cIMT. When categorizing into tertiles, a one-SD increase in fasting insulin and HOMA-IR were associated with 87 and 81% increases in the odds of higher categories of cIMT (both *P* < 0.05). However, a one-SD increase in QUICKI was associated with a 37% lower odds of higher categories of cIMT (*P* = 0.022). No significant associations were found among girls (Table 2).

**Table 2:** Associations of insulin metabolism markers with carotid intima media thickness according to sex

	Multiple linear regression		Multiple ordinal regression^*^	
	Standardized β	*P*-value	OR (95% CI)	*P*-value
Fasting insulin (μU/ml)				
Boys				
Unadjusted	0.299	< 0.001	1.49 (1.10–2.01)	0.009
Model 1^†^	0.351	< 0.001	1.87 (1.24–2.81)	0.003
Model 2^‡^	0.344	< 0.001	1.84 (1.22–2.79)	0.004
Girls				
Unadjusted	0.010	0.911	1.21 (0.93–1.58)	0.154
Model 1^†^	−0.090	0.344	0.93 (0.67–1.29)	0.672
Model 2^‡^	−0.116	0.242	0.89 (0.63–1.25)	0.506
HOMA-IR				
Boys				
Unadjusted	0.308	< 0.001	1.51 (1.12–2.04)	0.006
Model 1^†^	0.350	< 0.001	1.81 (1.22–2.70)	0.003
Model 2^‡^	0.343	< 0.001	1.80 (1.20–2.68)	0.004
Girls				
Unadjusted	−0.006	0.951	1.18 (0.91–1.55)	0.215
Model 1^†^	−0.096	0.314	0.91 (0.66–1.27)	0.601
Model 2^‡^	−0.122	0.217	0.87 (0.61–1.23)	0.425
QUICKI				
Boys				
Unadjusted	−0.288	< 0.001	0.82 (0.63–1.06)	0.126
Model 1^†^	−0.305	0.001	0.63 (0.42–0.93)	0.022
Model 2^‡^	−0.298	0.001	0.64 (0.43–0.95)	0.027
Girls				
Unadjusted	−0.034	0.704	0.70 (0.49–1.00)	0.051
Model 1^†^	0.051	0.581	0.96 (0.60–1.54)	0.859
Model 2^‡^	0.077	0.430	1.00 (0.61–1.64)	0.994

*HOMA-IR* homeostatic model assessment of insulin resistance, *QUICKI* quantitative insulin sensitivity check index

^*^Multiple ordinal regression was used with carotid intima media thickness as response with three ordered categories

^†^Adjusted for body fat mass percent, waist circumference, and physical activity

^‡^Additionally adjusted for Tanner stages

## Discussion

This study demonstrated that markers of insulin metabolism were independent predictors of cIMT in overweight and obese boys, but not in girls. Both fasting insulin and HOMA-IR were positively, and QUICKI negatively associated with cIMT. This relationship remained significant even after adjusting for obesity features such as body fat percentage and WC, or pubertal status (Tanner stages). This result not only highlights the robustness of the link between insulin resistance and cIMT as an independent risk factor for the development of atherosclerosis, but it clearly underscores how arterial abnormalities are demonstrable already at a very early age in overweight and obese boys.

A number of observational studies among adults have shown adverse structural changes in cIMT in the presence of cardiovascular (CVD) risk factors [17, 18]. However, data regarding subclinical CVD using imaging such as cIMT in pediatrics with overweight and obesity is limited and inconsistent. While some studies reported that increased cIMT was mediated in part by obesity [19], insulin sensitivity [20], type 2 diabetes [21], DBP [22], SBP, inflammatory markers [23], epicardial adipose tissue thickness [24], WC [22, 25], others found no associations between cIMT and insulin resistance [14, 26], prediabetes state [27], polycystic ovary syndrome [28], and inflammatory chemokines [29].

Our findings are in agreement with results of recent investigations where higher insulin levels and the presence of insulin resistance were adversely associated with carotid wall thickness among children and adolescents. Ryder et al. (2016) conducted a cross-sectional study on 252 children, aged between 8 and 20 years and in Tanner stages ranging from 2 to 5, and observed that cIMT was positively related to insulin resistance (measured by hyperinsulinemic euglycemic clamp) [10]. In addition, type 2 diabetes, a state of insulin resistance, was associated with higher cIMT in youth aged 10–24 years [21]. Furthermore, Atabek et al. observed in obese children aged between 8 and 18 years that QUICKI was independently associated with cIMT, but not HOMA-IR [30]. However, Reinehr et al. did not find any significant correlation between insulin level and cIMT among 96 obese children with a mean age of 11 years, after controlling for pubertal status and body fat mass [23]. Besides, there was no significant difference with regard to IMT between obese insulin resistant and obese not insulin resistant children aged 12 to 18 years, a fact that could be due to the β-error associated with the sample size [14]. Compared to most previous studies, our study had a larger sample size, narrower age range, and accounted for potential confounding variables (physical activity, body fat percentage, WC, and pubertal status). In contrast to these studies, our findings clearly indicate how markers of insulin metabolism are associated with carotid atherosclerosis, and we found this association to be independent of adiposity. These differences may be due to the fact that we considered an overweight to an obese pediatric population with a narrower BMI than most other studies. In addition, these differences may also be partly due to differences in the protocol used for measuring cIMT.

Another difference compared to previous studies is that we separated our participants by gender. This yielded the somewhat surprising result that the association between insulin-related variables and cIMT exists only among boys but not among girls. The reason for this finding is not clear; there is a difference between girls and boys regarding weight status and WC values. Lower obesity prevalence and WC among girls compared to these among boys may be an explanation to justify lack of association between insulin resistance and IMT in girls. It might also explain the higher susceptibility of boys to developing CVD and the fact that CVD events occur approximately 10 years earlier in life for men that for women [31]. Increased adiposity, in combination with higher glucose and insulin levels during childhood, has been linked to a higher carotid thickness in later life [12, 30]. The Bogalusa Heart Study found that the cumulative levels of high BMI in childhood was associated with adult cIMT in both sexes, even after controlling for adult BMI [32]. Increased body weight may interact with insulin level and sensitivity by affecting atherosclerosis.

Several studies illustrated an association between obesity and thicker cIMT among children and adolescents. The significance of this relation became marginal once the model applied to 100 children with BMI >95th percentile was adjusted for glucose [19]. Besides, Reinehr et al. revealed that impaired fasting glucose, along with BMI, was an independent determinant of cIMT in obese children. Their results indicate the importance of impaired glucose hemostasis for the arterial structure during childhood and even adulthood [12]. Furthermore, the linkage of insulin resistance and increased cIMT may be explained by the atherogenic pattern of lipoprotein particles and higher endothelial-derived ICAM, which are both present among obese insulin-resistant adolescents [14].

By surveying a large number of children and adolescents, we could observe associations with regard to gender. BIA measurements allowed us to adjust for adiposity (body fat percentage) instead of only BMI. Despite these strengths, our study also has some inherent limitations: the participants came from a narrowly localized geographic region (the southern districts of Tehran), which may limit generalizability; due to this study having been strictly observational, causality could be established; despite measurement of a number of confounders, residual confounding could not be ruled out. Assessing breast development among overweight and obese girls may be overestimated because of difficulties in distinguishing between glandular breast tissue and subcutaneous fat; however, in the current study, a well-trained woman endocrinologist was used to visual assessment of breast budding (Tanner stage 2 development) which minimized measurement error. And lastly, our interpretation of insulin resistance is predominantly based on the HOMA-IR and not on euglycaemic clamp studies, which was simply not possible given our study parameters.

## Conclusion

In conclusion, the current study showed that HOMA-IR and QUICKI, as a markers of insulin resistance and sensitivity, may serve as independent predictors of cIMT in overweight and obese boys, but not in girls. Our study provides biologically important information and further work is recommended to translate these findings into clinical practice for identifying high-risk children.

## Abbreviations

BIA: bioelectrical impedance
BMI: body mass index
CCA: common carotid artery
cIMT: Carotid intima media thickness
DBP: diastolic blood pressure
FBG: fasting blood glucose
HOMA-IR: homeostatic model assessment of insulin resistance
POM: proportional odds model
QUICKI: quantitative insulin sensitivity check index
SBP: systolic blood pressure
SD: standard deviation
WC: waist circumference
